# Classified Comparison and Cause Analysis of the Characteristics of Co‐Disabilities Among Older Adults With Physical Disability in China

**DOI:** 10.1002/hcs2.70088

**Published:** 2026-07-13

**Authors:** Yiran Wang, Xiaodong Zhang, Lu Tan, Chenyu Yan, Xiaoqian Yan, Wenwen Liu, Tianran Wang, Sijiu Wang, Wannian Liang

**Affiliations:** ^1^ Vanke School of Public Health Tsinghua University Beijing China; ^2^ T.H. Chan School of Public Health Harvard University Boston United States; ^3^ Department of Sociology People's Public Security University of China Beijing China; ^4^ School of Media and Communication Shanghai Jiao Tong University Shanghai China; ^5^ School of Nursing and Rehabilitation Shandong University Jinan China; ^6^ College of General Practice Southern University of Science and Technology Shenzhen China

**Keywords:** age‐standardized prevalence, co‐disability, older adult physical disability

## Abstract

**Background:**

The aim of the study is to investigate the characteristics of co‐disabilities among older adults with physical disabilities (PDs) in China in 1987 and 2006, and the correlation between other co‐occurring disabilities and the presence and severity of PD among the older adults.

**Methods:**

The study is a cross‐sectional comparative study. The data were derived from the first and second China National Sample Survey on Disability. Case counts and prevalence rates were used to compare the characteristics of PD and “co‐disabilities comprising PD and other disabilities” (CdPDD) in these two surveys. The binary logistic regression was used to analyze the correlation.

**Results:**

(1) From 1987 to 2006, the age‐standardized prevalence rates of PD, sole PD, and CdPDD among older adults in China all increased, and these increases were more pronounced in rural older adults. (2) In both years, co‐disabilities involving PD and hearing‐speech disability had the highest percentage distribution and age‐standardized prevalence rate, accounting for more than 60%, and this pattern was consistent in both urban and rural older adults. (3) Speech and intellectual disabilities were consistently associated with higher odds of PD and moderate‐to‐severe PD in both the overall sample and the urban–rural subgroup analyses. (4) Co‐disabilities involving PD and mental disability showed a marked increase over time in both urban and rural older adults, particularly for multiple‐CdPDD.

**Conclusions:**

PD and CdPDD among older adults in China increased markedly from 1987 to 2006, especially in rural areas. Hearing‐speech‐related co‐disabilities remained the dominant pattern, while mental‐related co‐disabilities increased most rapidly. The associations of different co‐occurring disability types with PD and moderate‐to‐severe PD varied. In the short term, priority should be given to identifying high‐risk co‐disability combinations and strengthening screening, referral, rehabilitation follow‐up, and continuity of care, particularly in rural areas. In the longer term, a more integrated co‐disability management framework is needed to better connect rehabilitation, mental health, primary healthcare, and long‐term care services. Greater use of the co‐disability rate indicator may also help guide priority setting and service planning.

## Graphical abstract

This study used prevalence comparisons and binary logistic regression to analyze the data from the First and Second China National Sample Survey on Disability conducted in 1987 and 2006, respectively. Co‐disability patterns among older adults with physical disability in China were examined, and the associations of other disability types with both the presence and severity of physical disability were identified.

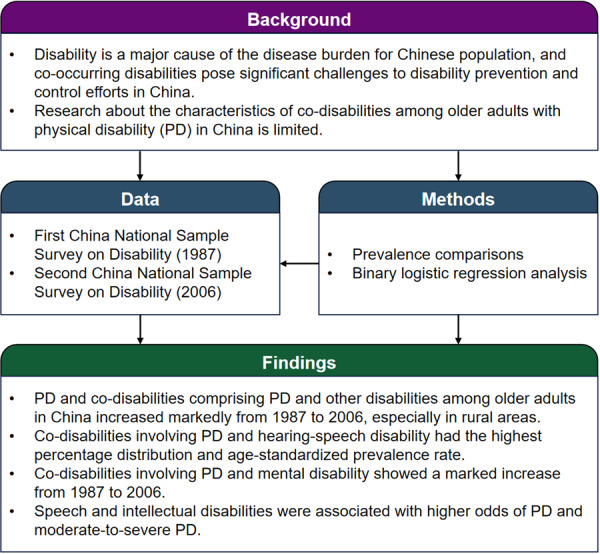

**Abbreviations**
CdPDD: co‐disabilities comprising PD and other disabilities
CI: confidence interval
OR: odds ratio
PD: physical disability

## 1 Background

Disability is a major cause of the disease burden for the Chinese population. On the one hand, there is a large number of people living with disabilities. It is projected that the population life expectancy at birth in China will increase to approximately 80.1 years in 2030 and 81.3 years in 2035 [1, 2]. However, it is also accompanied by an increase in the proportion of life expectancy lived with disability, which is expected to increase from 9.60% in 2015 to 14.54% in 2050 [3]. Specifically, disability life expectancy among older adults is expected to rise from 5.78 years in 2015 to 11.45 years in 2050 [3]. According to the results of the 2006 Second China National Sample Survey on Disability, among the seven categories of disabilities (sole visual, hearing, speech, physical, intellectual, mental disabilities, and multiple disabilities), individuals with sole physical disability (PD) account for approximately 29.07% [4], making it the largest category of disabilities in China. Between 1987 and 2006, the prevalence of PD in China rose across different age groups, with the largest increase occurring among older adults [5]. On the other hand, there arises the problem of co‐occurring disabilities. According to the results of the survey in 2006, 3.93% of older adults in China have co‐occurring disabilities, second to the percentage of the elderly with only hearing, visual, or PDs. Co‐occurring disabilities pose significant challenges to disability prevention and control efforts in China because of their complex etiological mechanisms and the greater difficulty of effective intervention. An increasing number of researchers have reached a consensus that rehabilitation methods and technologies designed solely for single disabilities may not adequately meet the complex needs of individuals with concurrent disabilities [6].

With the continued advancement of health policies such as Universal Health Coverage, disability among older adults has attracted increasing global attention. Accordingly, a growing body of research has examined functional limitations, rehabilitation needs and strategies, and disability trajectories in aging populations [6, 7, 8, 9, 10]. In China, previous studies have examined the prevalence and trends of co‐disabilities in children with intellectual disabilities [11] and older adults with hearing‐speech disabilities [12], with only limited evidence on co‐occurring mental disability among older adults with PD [13]. Research on co‐disabilities among older adults still remains limited.

Against this background, the present study conducted a comparative analysis of co‐occurring disabilities among older adults with PD using data from two nationally representative disability surveys conducted in China in 1987 and 2006. Specifically, this study aimed to address existing research gaps by examining long‐term changes in the prevalence and composition of co‐disabilities among older adults with PD, as well as the associations of different co‐occurring disability types with the presence of PD and moderate‐to‐severe PD. By doing so, this study seeks to enrich the literature on co‐occurring disabilities among older adults.

Additionally, it is pertinent to current national policy priorities in China, including the Healthy China 2030 initiative and the national strategy for actively addressing population aging. As these policy frameworks place increasing emphasis on disability prevention, rehabilitation, and integrated services for older adults, the present study provides empirical evidence that may help inform related policy development, particularly in identifying high‐risk disability combinations and supporting more targeted prevention, rehabilitation, and care.

## 2 Methods

### 2.1 Data Source

The data for this study were sourced from the First China National Sample Survey on Disability conducted in 1987 and the Second China National Sample Survey on Disability conducted in 2006. Both surveys adopted stratified, multi‐stage, cluster, and probability proportional sampling methods. The first survey examined a total of 1,579,316 people from 369,448 households in 29 provinces (districts and cities), 424 counties, and 3169 small communities at 0:00 on April 1, 1987. The second survey included a total of 2,526,145 people from 771,797 households in 31 provinces (districts and cities), 734 counties, and 5974 small communities at 0:00 on April 1, 2006 [14, 15]. The final analytical samples included 140,042 and 354,859 older adults aged 60 years and above from the 1987 and 2006 surveys, respectively. The quality of the survey data is authentic and reliable, adequately reflecting the actual conditions of the disabled population in China [16]. In addition, the original survey protocol included standardized quality assurance procedures during fieldwork, including household‐based investigation, individual screening and physician assessment, self‐checks, mutual checks, discussion‐based checks, logical verification of completed forms, and post‐survey quality audits. Before analysis, we also checked variable coding, category consistency, and basic range and logical consistency of key variables.

### 2.2 Core Concepts

#### 2.2.1 PD

PD refers to impairment of body movement functions due to structural or functional damage to the motor system, resulting in limb deficiency, paralysis of limbs or trunk, deformities, leading to varying degrees of loss of physical movement function and activity or participation limitations [17].

#### 2.2.2 Co‐Occurring Disabilities/Co‐Disabilities

The term “sole PD” refers to cases in which individuals have only PD without any other types of disabilities, while “co‐occurring disabilities” or “co‐disabilities” describe situations where individuals have PD along with one or more additional types of disabilities. Co‐disabilities comprising PD and other disabilities (CdPDD) are further categorized into dual‐CdPDD and multiple‐CdPDD, with dual‐CdPDD referring to PD‐individuals along with one other type of disability, and multiple‐CdPDD referring to PD‐individuals along with two or more other types of disabilities [11].

### 2.3 Measure and Statistical Analysis

For the descriptive analysis, we primarily examined CdPDD among older adults using data from the 1987 and 2006 surveys, reporting indicators such as case counts, percentage distributions, prevalence rates, and temporal trends for the overall sample as well as for urban and rural subgroups. To ensure comparability across survey years and between urban and rural areas, this study age‐standardized the prevalence rates using older adult population data from the Fifth National Population Census conducted in 2000. It should be noted that there were slight differences in disability classification between 1987 and 2006. In the 1987 survey, disabilities were classified into five categories: visual, hearing‐speech, physical, intellectual, and mental disabilities. In the 2006 survey, hearing disability and speech disability were listed separately. However, the overall disability classification framework and the assessment criteria for the major disability types were broadly consistent with international standards and were generally comparable [18, 19]. To ensure comparability under a unified classification scheme, this study followed the standards established in 1987 and combined hearing disability and speech disability into a single category of hearing‐speech disability.

For the regression analysis, binary logistic regression models were constructed using only the 2006 data to examine the associations in the overall sample as well as in urban and rural subgroups. The dependent variables were whether an individual has PD and whether an individual has a moderate‐to‐severe PD. For models in which the dependent variable was the presence of PD, the independent variable was the type of co‐occurring non‐PD disability, categorized into five groups: visual disability as the only non‐PD disability, hearing disability as the only non‐PD disability, speech disability as the only non‐PD disability, intellectual disability as the only non‐PD disability, and mental disability as the only non‐PD disability; individuals with no non‐PD disability served as the reference group. For models in which the dependent variable was moderate‐to‐severe PD, the independent variable was the type of co‐occurring disability among individuals with PD, categorized into five groups: PD with visual disability only, PD with hearing disability only, PD with speech disability only, PD with intellectual disability only, and PD with mental disability only; individuals with PD only served as the reference group. To obtain the net effects of having visual, hearing, speech, intellectual, or mental disabilities on the presence and severity of PD, samples with two or more other types of disabilities were excluded from the regression analysis [11].

The control variables included gender (male or female), age, place of residence (urban or rural), region (western, eastern, central, or northeastern China), ethnicity (Han or ethnic minority), and educational attainment (no formal education, primary school, junior high school, senior high school, technical secondary school, junior college, bachelor's degree, and postgraduate education). These control variables were selected based on theoretical considerations and prior empirical studies. First, the social determinants of health framework points out that health outcomes are shaped not only by demographic and biological characteristics, such as age and gender, but also by broader socioeconomic factors, such as resident status, region, ethnicity, and educational attainment [20]. Second, cumulative disadvantage theory proposes that social and economic inequality experienced in the earlier stages of life could accumulate over time, thus resulting in health vulnerability in the later stages of life [21]. Third, the socio‐ecological model suggests that health outcomes are shaped by multiple layers of factors, including individual, interpersonal, organizational, community, and policy‐level factors [22]. The inclusion of these control variables is also consistent with existing studies about disability and aging in the population of China [23, 24, 25]. Although the 2006 survey collected information on rehabilitation‐related service utilization and self‐reported service needs, these variables were not included in the primary regression models because they were collected only among persons who had already been identified as having disabilities, which limits their comparability across analytic groups.

PD severity in 2006 surveys was classified into four levels according to the official disability assessment framework. This grading system was based primarily on the extent to which a person could perform activities of daily living independently in the absence of rehabilitation aids or assistive devices. Functional mobility was assessed through sitting upright, standing, walking, and writing, while self‐care ability was assessed through washing, dressing, eating, and toileting. Based on the total score, PD was categorized into four levels: Level 1 (0–4.5), indicating complete dependence in activities of daily living and representing the most severe disability; Level 2 (5–8.5), indicating substantial dependence and severe disability; Level 3 (9–12.5), indicating partial independence and moderate disability; and Level 4 (13–16), indicating basic independence and mild disability. In this study, for analytical purposes, Level 4 was categorized as mild disability, whereas Levels 1–3 were combined as moderate‐to‐severe disability. This approach is also consistent with previous studies using the same survey data [11, 26].

Additionally, no imputation was performed for missing data. In the 2006 survey, there were no missing values for the main variables used in this study. In the 1987 survey, missingness in the main variables was minimal, with only seven cases missing information on urban–rural residence. Given the negligible extent of missing data, no additional missing‐data treatment was applied. Records with missing values were excluded only from the relevant descriptive analyses. All analyses were conducted using Stata 18.0 (StataCorp LLC, College Station, United States), with the significance level set at 0.01.

## 3 Results

### 3.1 Prevalence of PD and Co‐Disabilities Among Older Adults

#### 3.1.1 Prevalence of PD Among Older Adults

Using the age structure of the population aged 60 years and above from China's Fifth Population Census as the standard population, we calculated the age‐standardized prevalence rates of PD in 1987 and 2006. Over the 20‐year period, the age‐standardized prevalence rate of PD among older adults increased from 365/10,000 to 758/10,000, representing a rise of 107.67%. The age‐standardized prevalence rate of sole PD increased from 240/10,000 to 577/10,000, corresponding to a 140.42% increase. Meanwhile, the age‐standardized prevalence rate of CdPDD increased from 125/10,000 to 180/10,000, representing a 44.00% increase (Table 1).

**Table 1:** Prevalence rate of PD among older adults in 1987 and 2006.

Sample	Type of PD	1987 (*N* = 140,042)			2006 (*N* = 354,859)			Change in age‐standardized prevalence rate (1987–2006)/%
		*n*	Prevalence rate/10,000	Age‐standardized prevalence rate^a^/10,000	*n*	Prevalence rate/10,000	Age‐standardized prevalence rate^a^/10,000	
Overall	All PD	4952	354	365	28,758	810	758	107.67
	Sole PD	3324	237	240	21,470	605	577	140.42
	CdPDD	1628	116	125	7288	205	180	44.00
	Dual‐CdPDD	1213	87	92	5714	161	143	55.43
	Multiple‐CdPDD	415	30	33	1574	44	38	15.15
		1987 (*N* = 43,906^b^)			2006 (*N* = 122,893)			
Urban	All PD	1713	390	403	9056	737	681	68.98
	Sole PD	1154	263	267	6658	542	511	91.39
	CdPDD	559	127	136	2398	195	170	25.00
	Dual‐CdPDD	405	92	98	1836	149	131	33.67
	Multiple‐CdPDD	154	35	38	562	46	39	2.63
		1987 (*N* = 96,129^b^)			2006 (*N* = 231,966)			
Rural	All PD	3238	337	347	19,702	849	799	130.26
	Sole PD	2170	226	228	14,812	639	613	168.86
	CdPDD	1068	111	120	4890	211	186	55.00
	Dual‐CdPDD	807	84	89	3878	167	149	67.42
	Multiple‐CdPDD	261	27	30	1012	44	37	23.33

Abbreviations: CdPDD, co‐disabilities comprising PD and other disabilities; PD, physical disability.

^a^ Age‐standardized prevalence rates were calculated using data on older adults from China's Fifth National Population Census conducted in 2000.

^b^ Because seven observations had missing urban–rural information in the 1987 data set, seven records were excluded when calculating the age‐standardized prevalence separately for urban and rural areas. As a result, the combined number of rural and urban observations was seven fewer than the total overall sample.

Urban–rural stratified analyses showed that the age‐standardized prevalence rates of PD also increased in both urban and rural older adults between 1987 and 2006, but the increase was more pronounced in rural areas. Among urban older adults, the age‐standardized prevalence rate of all PD increased from 403/10,000 to 681/10,000, representing a rise of 68.98%; the corresponding rates for sole PD, CdPDD, dual‐CdPDD, and multiple‐CdPDD increased from 267/10,000 to 511/10,000 (91.39%), from 136/10,000 to 170/10,000 (25.00%), from 98/10,000 to 131/10,000 (33.67%), and from 38/10,000 to 39/10,000 (2.63%), respectively (Table 1).

Among rural older adults, the age‐standardized prevalence rate of all PD increased from 347/10,000 to 799/10,000, representing a rise of 130.26%; the corresponding rates for sole PD, CdPDD, dual‐CdPDD, and multiple‐CdPDD increased from 228/10,000 to 613/10,000 (168.86%), from 120/10,000 to 186/10,000 (55.00%), from 89/10,000 to 149/10,000 (67.42%), and from 30/10,000 to 37/10,000 (23.33%), respectively (Table 1).

Overall, from 1987 to 2006, the age‐standardized prevalence rates of PD, sole PD, and CdPDD among older adults in China all increased, and these increases were more pronounced in rural older adults.

#### 3.1.2 Co‐Disabilities Comprising PD and Other Disabilities Among Older Adults

Analysis of CdPDD showed that, in both 1987 and 2006, among older adults with dual‐CdPDD or multiple‐CdPDD, combinations of PD and hearing‐speech disability had the highest percentage distributions and age‐standardized prevalence rates, followed by combinations involving visual, intellectual, and mental disabilities. Co‐disabilities involving PD and hearing‐speech disability or visual disability together accounted for approximately 90% of all CdPDD cases. From 1987 to 2006, co‐disabilities involving mental disability showed the most pronounced increase in age‐standardized prevalence rates (Table 2).

**Table 2:** Co‐disability types comprising PD and other disabilities among older adults in 1987 and 2006.

Sample	Co‐disability types	1987 (*N* = 140,042)				2006 (*N* = 354,859)				Change in age‐standardized prevalence rate (1987–2006)/%
		*n*	Percentage distribution/%	Prevalence rate/10,000	Age‐standardized prevalence rate^a^/10,000	*n*	Percentage distribution/%	Prevalence rate/10,000	Age‐standardized prevalence rate^a^/10,000	
Overall	*Dual‐CdPDD*									
	Physical + visual	336	28	24	26	1650	29	46	41	57.69
	Physical + hearing‐speech	754	62	54	57	3626	63	102	90	57.89
	Physical + intellectual	108	9	8	8	243	4	7	6	−25.00
	Physical + mental	15	1	1	1	195	3	5	5	400.00
	*Multiple‐CdPDD*									
	Physical + visual + other	296	34	21	24	963	28	27	22	−8.33
	Physical + hearing‐speech + other	372	42	27	30	1397	40	39	33	10.00
	Physical + intellectual + other	191	22	14	15	641	18	18	16	6.67
	Physical + mental + other	18	2	1	1	471	14	13	12	1100.00
		1987 (*N* = 43,906^b^)				2006 (*N* = 122,893)				
Urban	*Dual‐CdPDD*									
	Physical + visual	100	25	23	25	397	22	32	28	12.00
	Physical + hearing‐speech	248	61	56	60	1286	70	105	92	53.33
	Physical + intellectual	51	13	12	12	82	4	7	6	−50.00
	Physical + mental	6	1	1	1	71	4	6	5	400.00
	*Multiple‐CdPDD*									
	Physical + visual + other	99	30	23	26	248	19	20	17	−34.62
	Physical + hearing‐speech + other	137	42	31	34	481	38	39	33	−2.94
	Physical + intellectual + other	83	25	19	20	319	25	26	23	15.00
	Physical + mental + other	9	3	2	2	227	18	18	16	700.00
Rural	*Dual‐CdPDD*	1987 (*N* = 96,129^b^)				2006 (*N* = 231,966)				
	Physical + visual	236	29	25	27	1253	32	54	48	77.78
	Physical + hearing‐speech	505	63	53	56	2340	60	101	89	58.93
	Physical + intellectual	57	7	6	6	161	4	7	7	16.67
	Physical + mental	9	1	1	1	124	3	5	5	400.00
	*Multiple‐CdPDD*									
	Physical + visual + other	197	36	20	23	715	33	31	25	8.70
	Physical + hearing‐speech + other	235	43	24	28	916	42	39	33	17.86
	Physical + intellectual + other	108	20	11	12	322	15	14	13	8.33
	Physical + mental + other	9	2	1	1	244	11	11	9	800.00

Abbreviations: CdPDD, co‐disabilities comprising PD and other disabilities; PD, physical disability.

^a^ Age‐standardized prevalence rates were calculated using data on older adults from China's Fifth National Population Census conducted in 2000.

^b^ Because seven observations had missing urban–rural information in the 1987 data set, seven records were excluded when calculating the age‐standardized prevalence separately for urban and rural areas. As a result, the combined number of rural and urban observations was seven fewer than the total overall sample.

Urban–rural stratified analyses showed that, in both urban and rural older adults, co‐disabilities involving PD and hearing‐speech disability had the highest percentage distributions and age‐standardized prevalence rates among dual‐CdPDD in both 1987 and 2006. Among urban older adults, the percentage distribution of physical + hearing‐speech disability increased from 61% to 70%, and its age‐standardized prevalence rate increased from 60/10,000 to 92/10,000. Among rural older adults, the corresponding percentage distribution changed from 63% to 60%, while the age‐standardized prevalence rate increased from 56/10,000 to 89/10,000. For multiple‐CdPDD, co‐disabilities involving PD, hearing‐speech disability, and other disabilities also remained the most common type in both urban and rural areas, accounting for 42% and 38% in urban areas and 43% and 42% in rural areas in 1987 and 2006, respectively (Table 2).

From 1987 to 2006, mental‐related co‐disabilities showed the most marked increase in both settings, especially among multiple‐CdPDD. In urban older adults, the age‐standardized prevalence rate of physical + mental disability increased from 1/10,000 to 5/10,000, and that of physical + mental + other disabilities increased from 2/10,000 to 16/10,000. In rural older adults, the corresponding rates increased from 1/10,000 to 5/10,000 and from 1/10,000 to 9/10,000, respectively (Table 2).

Overall, hearing‐speech‐related co‐disabilities remained the dominant pattern among both urban and rural older adults, and mental‐related co‐disabilities showed a particularly pronounced increase over time.

### 3.2 The Relationship Between PD and Co‐Disabilities With Other Disabilities Among Older Adults

After excluding older adults with multiple‐CdPDD, we performed logistic regression analysis, which showed distinct associations between specific co‐occurring disabilities and PD. After adjustment for six sociodemographic variables, speech disability (odds ratio [OR] = 23.128, 95% confidence interval [CI] = 20.693–25.848) and intellectual disability (OR = 2.396, 95% CI = 2.079–2.763) were associated with higher odds of having PD, whereas visual disability (OR = 0.917, 95% CI = 0.869–0.968) and hearing disability (OR = 0.766, 95% CI = 0.733–0.801) were associated with lower odds of having PD. No significant association was observed for mental disability (Table 3).

**Table 3:** The relationship between PD and co‐disabilities with other disabilities among older adults (2006).

Sample	Groups	OR	S.E.	*z*	95% CI
Overall	No non‐PD disability	1.000	—	—	—
	Visual disability as the only non‐PD disability	0.91*	0.025	−3.16	0.869–0.968
	Hearing disability as the only non‐PD disability	0.766*	0.017	−11.75	0.733–0.801
	Speech disability as the only non‐PD disability	23.128*	1.312	55.36	20.693–25.848
	Intellectual disability as the only non‐PD disability	2.396*	0.174	12.04	2.079–2.763
	Mental disability as the only non‐PD disability	1.010	0.076	0.12	0.870–1.171
Urban	No non‐PD disability	1.000	—	—	—
	Visual disability as the only non‐PD disability	1.037	0.058	0.65	0.930–1.156
	Hearing disability as the only non‐PD disability	0.877*	0.035	−3.28	0.810–0.948
	Speech disability as the only non‐PD disability	32.395*	2.989	37.69	27.035–38.817
	Intellectual disability as the only non‐PD disability	3.231*	0.418	9.06	2.507–4.163
	Mental disability as the only non‐PD disability	1.262	0.160	1.84	0.985–1.619
Rural	No non‐PD disability	1.000	—	—	—
	Visual disability as the only non‐PD disability	0.883*	0.028	−3.92	0.830–0.940
	Hearing disability as the only non‐PD disability	0.724*	0.020	−11.76	0.686–0.764
	Speech disability as the only non‐PD disability	18.640*	1.349	40.43	16.176–21.479
	Intellectual disability as the only non‐PD disability	2.117*	0.187	8.51	1.781–2.516
	Mental disability as the only non‐PD disability	0.900	0.085	−1.11	0.748–1.083

Abbreviations: —, not applicable; CI, confidence interval; OR, odds ratio; PD, physical disability; S.E., standard error.

* *p* < 0.01.

Urban–rural stratified analyses revealed broadly similar patterns of association. Among urban older adults, hearing disability was associated with lower odds of PD (OR = 0.877, 95% CI = 0.810–0.948), whereas speech disability (OR = 32.395, 95% CI = 27.035–38.817) and intellectual disability (OR = 3.231, 95% CI = 2.507–4.163) were associated with higher odds of PD. No significant association was observed for visual disability (OR = 1.037, 95% CI = 0.930–1.156) or mental disability (OR = 1.262, 95% CI = 0.985–1.619) in urban older adults. Among rural older adults, visual disability (OR = 0.883, 95% CI = 0.830–0.940) and hearing disability (OR = 0.724, 95% CI = 0.686–0.764) were associated with lower odds of PD, whereas speech disability (OR = 18.640, 95% CI = 16.176–21.479) and intellectual disability (OR = 2.117, 95% CI = 1.781–2.516) were associated with higher odds of PD. Mental disability was not significantly associated with the odds of PD in rural older adults (OR = 0.900, 95% CI = 0.748–1.083) (Table 3).

Overall, speech and intellectual disabilities were consistently associated with higher odds of PD in both urban and rural older adults.

### 3.3 The Relationship Between the Severity of PD and Co‐Disabilities With Other Disabilities Among Older Adults

The logistic regression analysis showed that, after adjusting for six sociodemographic variables, hearing disability was associated with lower odds of moderate‐to‐severe PD (OR = 0.710, 95% CI = 0.651–0.775), whereas speech disability (OR = 7.464, 95% CI = 6.191–9.000), intellectual disability (OR = 3.527, 95% CI = 2.644–4.704), and mental disability (OR = 1.903, 95% CI = 1.424–2.542) were associated with higher odds of moderate‐to‐severe PD. Visual disability was not significantly associated with the odds of moderate‐to‐severe PD (Table 4).

**Table 4:** The relationship between moderate‐to‐severe PD and co‐disabilities with other disabilities among older adults (2006).

Sample	Groups	OR	S.E.	*z*	95% CI
Overall	PD only	1.000	—	—	—
	PD with visual disability only	0.977	0.051	−0.44	0.881–1.083
	PD with hearing disability only	0.710*	0.032	−7.71	0.651–0.775
	PD with speech disability only	7.464*	0.712	21.07	6.191–9.000
	PD with intellectual disability only	3.527*	0.518	8.57	2.644–4.704
	PD with mental disability only	1.903*	0.281	4.35	1.424–2.542
Urban	PD only	1.000	—	—	—
	PD with visual disability only	0.858	0.091	−1.44	0.696–1.057
	PD with hearing disability only	0.632*	0.050	−5.84	0.542–0.737
	PD with speech disability only	9.406*	1.558	13.53	6.798–13.015
	PD with intellectual disability only	3.724*	1.006	4.87	2.193–6.324
	PD with mental disability only	1.817	0.448	2.42	1.120–2.947
Rural	PD only	1.000	—	—	—
	PD with visual disability only	1.014	0.061	0.23	0.901–1.142
	PD with hearing disability only	0.745*	0.040	−5.46	0.670–0.828
	PD with speech disability only	6.477*	0.761	15.91	5.145–8.154
	PD with intellectual disability only	3.434*	0.603	7.02	2.434–4.846
	PD with mental disability only	1.950*	0.359	3.62	1.359–2.799

Abbreviations: —, not applicable; CI, confidence interval; OR, odds ratio; PD, physical disability; S.E., standard error.

* *p* < 0.01.

Urban–rural stratified analyses showed that, among urban older adults, hearing disability was associated with lower odds of moderate‐to‐severe PD (OR = 0.632, 95% CI = 0.542–0.737), whereas speech disability (OR = 9.406, 95% CI = 6.798–13.015) and intellectual disability (OR = 3.724, 95% CI = 2.193–6.324) were associated with higher odds. Visual and mental disabilities were not significantly associated with moderate‐to‐severe PD in urban areas. Among rural older adults, hearing disability was likewise associated with lower odds of moderate‐to‐severe PD (OR = 0.745, 95% CI = 0.670–0.828), whereas speech disability (OR = 6.477, 95% CI = 5.145–8.154), intellectual disability (OR = 3.434, 95% CI = 2.434–4.846), and mental disability (OR = 1.950, 95% CI = 1.359–2.799) were associated with higher odds of moderate‐to‐severe PD. Visual disability was not significantly associated with moderate‐to‐severe PD in rural areas (Table 4).

Overall, speech and intellectual disabilities were consistently associated with higher odds of moderate‐to‐severe PD in both urban and rural older adults.

## 4 Discussion

### 4.1 Main Findings

First, from 1987 to 2006, the age‐standardized prevalence rates of PD, sole PD, and CdPDD among older adults in China all increased, and these increases were more pronounced in rural older adults. Second, in both years, co‐disabilities involving PD and hearing‐speech disability showed the highest percentage distribution and age‐standardized prevalence rate, accounting for more than 60%, and this clustering pattern was consistently observed in both urban and rural older adults. Third, speech and intellectual disabilities were consistently associated with higher odds of PD and moderate‐to‐severe PD in both the overall sample and the urban–rural subgroup analyses. Fourth, co‐disabilities involving PD and mental disability increased markedly over time in both urban and rural older adults, particularly for multiple‐CdPDD.

### 4.2 Interpretation of Findings

#### 4.2.1 The Increase of PD and CdPDD

The overall increase in PD and CdPDD may reflect both the growing disability burden in an aging society and the expansion of the older disabled population in China [5, 16, 27]. Since the beginning of the 21st century, China has entered a period of rapid population aging, and older adults have accounted for an increasingly large share of all persons with disabilities [5, 27]. At the same time, industrialization, urbanization, population mobility, traffic injuries, occupational risks, and environmental exposures may also have contributed to the overall increase in disability burden [27]. In addition, compared with the 1987 survey, the 2006 survey broadened the inclusion of several mild forms of PD, including unilateral total loss of the thumb, loss above the metatarsophalangeal joint of 1 ft, complete loss or loss of function of both toes, and dwarfism (height not exceeding 130 cm), which may also have increased the observed prevalence of PD [5, 16]. The more pronounced increase observed in rural older adults is particularly noteworthy. This pattern may reflect not only population aging in rural areas, but also structural disadvantages in access to healthcare, rehabilitation, long‐term care, and social support services in rural settings. Rural older adults with disabilities may face greater unmet care needs and fewer opportunities for early rehabilitation, which could contribute to the faster increase in both PD and CdPDD [28, 29, 30, 31, 32, 33, 34, 35].

#### 4.2.2 The Co‐Occurrence of PD and Other Disabilities

The overlap in risk factors across certain disability types may increase the likelihood of co‐disabilities. The clustering of PD with hearing‐speech disability, together with the substantially higher odds of PD among older adults with co‐occurring speech or intellectual disability, may suggest a closer relationship between these disability types and PD than other disabilities. In contrast, the odds of PD were significantly lower among older adults with visual or hearing disability, while no statistically significant association was observed for mental disability. One possible explanation for this pattern is that some disability types share more closely related etiological pathways with PD than others. Brain disorders are important causes of physical, intellectual, and speech disabilities, and in older adults, both PD and speech disability are often linked to cerebrovascular disease [36]. An epidemiological study of disability caused by cerebrovascular diseases also showed that individuals with speech disability and PD attributable to cerebrovascular conditions share similar demographic characteristics [37]. By contrast, the major causes of visual, hearing, and mental disabilities differ substantially from those of PD. According to the Second China National Sample Survey on Disability conducted in 2006, visual disability among older adults is mainly attributable to cataracts (55.10%) and retinal and pigmentary disorders (10.81%); hearing disability is primarily associated with presbycusis (64.99%) and noise or blast exposure (6.51%); and mental disability is mainly related to schizophrenia (33.97%) and dementia (33.00%).

#### 4.2.3 The Increase of Co‐Disabilities Involving PD and Mental Disability

The rising prevalence of mental disability has likely contributed to the increased prevalence of co‐disabilities involving PD and mental disability [38, 39, 40]. The 2006 national disability survey showed that the prevalence of mental disability in China had reached 6.3‰, accounting for 9.97% of the total disabled population, representing an increase of 4.46 per 1000 population in prevalence and 6.21 percentage points in proportion compared with 1987 [39]. As a major contributor to mental disability, the age‐standardized prevalence of schizophrenia increased by 3.1% in 2019 compared with 1990 [40]. In the context of rapid social transition, population aging, changing family structure, and increasing psychosocial stress, mental disability may have become more likely to be embedded in more complex forms of disability burden [38, 40]. In addition to the increase in mental disability prevalence itself, the substantial rise in co‐disabilities involving PD and mental disability may also reflect broader structural determinants during China's transition period. (1) For much of this period, mental health services in China remained relatively limited and unevenly distributed, with shortages in workforce and service capacity and incomplete integration of mental healthcare into general health services, rehabilitation, and primary care [41, 42]. For older adults, these service gaps may be particularly consequential because delayed detection, fragmented follow‐up, and insufficient rehabilitation continuity can make mental and physical impairments more likely to coexist and remain insufficiently managed. (2) Stigma surrounding mental illness and low mental health literacy may also have shaped the observed pattern. Previous studies in China have shown that stigma, reluctance to disclose mental illness, family opposition, self‐reliance, and limited accessibility remain important barriers to professional mental health help‐seeking [43]. Among older adults, these barriers may be further compounded by generational attitudes toward mental illness, reduced help‐seeking motivation, and dependence on family members for access to care. (3) Changes in family structure and social support systems during China's broader socioeconomic transition may also be relevant. Prior research suggests that family structure and family functioning are closely associated with mental health during China's transition [44]. For older adults in particular, declining household size, out‐migration of adult children, and social isolation may increase both psychological vulnerability and the risk that mental disability becomes entangled with PD.

### 4.3 Support From Related Literature and Interpretive Considerations

Recent studies continue to suggest that co‐disability remains a substantial and policy‐relevant problem. For example, recent research on older adults with hearing‐speech disability in China has shown that co‐disabilities remain common and structurally differentiated across disability types, while broader analyses of mental disability in China indicate that social‐context changes continue to shape mental disability patterns and may contribute to the persistence of complex co‐occurring disability burdens [12, 38]. In addition, recent international rehabilitation literature has emphasized that multiple disabilities generate needs that are more complex than those associated with single disabilities and often cannot be adequately addressed by conventional single‐disability rehabilitation models [7]. Taken together, these more recent findings do not directly update the prevalence estimates reported in this study, but they do provide support for some results identified in our analysis, particularly the clustering of hearing‐speech‐related co‐disabilities and the marked increase in mental‐related co‐disabilities.

Importantly, our regression results should be understood as identifying cross‐sectional associations rather than causal effects. The regression analysis in this study is based on cross‐sectional data, which does not allow us to determine the temporal ordering of co‐occurring disabilities. Therefore, we cannot establish whether another disability preceded PD, followed it, or developed concurrently. This limitation is important for interpreting the observed associations. In particular, the elevated odds ratios observed for speech and intellectual disabilities should not be interpreted as evidence that these disabilities cause PD; they may instead reflect shared etiological pathways or clustering of impairments.

Additionally, the most recent survey data used in this study were collected in 2006. Since then, China has undergone substantial socioeconomic transformation, healthcare system reform, and demographic transition. These changes—including the expansion of primary healthcare, the gradual development of long‐term care service arrangements, changes in family structure, and the growing emphasis on integrated care for older adults—may have influenced the prevalence and distribution patterns of co‐disability among older adults. Therefore, the prevalence estimates reported in this study should not be interpreted as a direct description of the current situation. Rather, they are better understood as evidence on long‐term structural patterns and historical change based on nationally representative disability survey data. Nevertheless, the data sets used in this study remain among the most valuable nationally representative data sources currently available for disability research in China [45, 46]. Notably, the 2006 survey is still the latest nationally representative disability survey conducted by the government in China. More importantly, these data sets are among the only national data sets that simultaneously cover clinically validated assessments of visual, hearing, speech, physical, intellectual, and mental disabilities under unified diagnostic criteria [14, 15, 16, 19]. They therefore provide uniquely valuable baseline evidence for examining long‐term trends in disability and co‐disability patterns.

### 4.4 Policy Implications

From an international comparative perspective, China's co‐disability patterns show several notable features, including the persistent clustering of co‐disabilities involving physical and hearing‐speech disability, the marked increase in mental‐related co‐disabilities, and the more pronounced rise observed in rural older adults. These findings suggest that co‐disability among older adults in China is shaped not only by disability‐specific mechanisms, but also by broader structural conditions such as rural‐urban disparities. In this context, international evidence provides useful guidance for implementation. The World Health Organization's *Community‐based rehabilitation: CBR guidelines* emphasize person‐centered, community‐based, and multisectoral approaches [47], while *Rehabilitation 2030* and *Rehabilitation in Health Systems: Guide for Action* stress integrating rehabilitation into health systems through coordinated planning, referral pathways, workforce development, and monitoring [48, 49]. In China, these principles could be aligned with current reforms such as *Healthy China 2030*, the hierarchical diagnosis and treatment system, and medical‐elderly care integration [50].

In the short term, greater attention should be given to the early identification of high‐risk co‐disability combinations and to strengthening screening, referral, rehabilitation follow‐up, and continuity of care for older adults with complex needs, particularly in rural areas. In practice, within the National Basic Public Health Service Program, routine health examinations and electronic health records could be used to identify older adults with co‐disabilities earlier and to support individualized guidance and referral [51]. Primary healthcare institutions and family doctor teams could support early screening, referral, and follow‐up [52], while higher‐level hospitals and rehabilitation institutions could provide referral‐based specialist assessment and more complex rehabilitation services. Additionally, the long‐term care insurance system may offer an additional pathway for older adults with severe functional limitations by linking disability assessment with nursing support, rehabilitation services, and long‐term care coordination [52, 53].

In the longer term, it is necessary to build a more integrated co‐disability management framework that better connects rehabilitation, mental health, primary healthcare, and long‐term care services. Current policy initiatives in China, including primary healthcare reform, family doctor services, and medical‐elderly care integration, may provide practical entry points for incorporating co‐disability prevention and management into routine service delivery. At the same time, it is necessary to make greater use of the co‐disability rate indicator. Our findings suggest that the co‐disability rate may serve as a practical indicator for policy and service management and evaluation. In healthcare planning, it can help define the priority combinations of disabilities and guide the development of integrated service pathways for screening, referral, rehabilitation, and follow‐up. In resource allocation, it can support more targeted distribution of rehabilitation, mental health, and long‐term care resources by highlighting high‐burden groups. In program evaluation, it can be used to monitor trends in co‐disability burden, assess whether interventions are reducing complex disability needs, and compare the effectiveness of different service delivery models. The use of the co‐disability rate indicator requires caution. It should be applied under standardized and comparable disability definitions, and interpreted together with indicators of disability severity, rehabilitation service availability, and local population structure. The indicator may be affected by differences in diagnostic capacity, case identification, reporting practices, and survey coverage.

### 4.5 Limitations and Future Research

This study has some limitations. First, the data used in this study were collected in 2006, which may limit the direct applicability of the findings to the current context. Over the past two decades, China has experienced substantial socioeconomic changes, healthcare reforms, and demographic transitions that may have influenced disability distribution patterns. As a result, the prevalence estimates reported in this study may not fully reflect the current situation. Moreover, many recent studies on older adults use indicators such as functional impairment, activities of daily living limitations, or care needs rather than the disability classification system adopted in the national disability surveys. Therefore, more recent evidence can serve mainly as contextual support rather than as directly comparable estimates. Future research using newer nationally or regionally representative data is needed to validate and extend these findings. Second, this study employed cross‐sectional data in the regression analysis, making it difficult to determine the sequence of onset for various disabilities. Consequently, this research is limited to examining correlations, and further cohort analyses are needed in subsequent studies.

## 5 Conclusions

PD and CdPDD among older adults in China increased markedly from 1987 to 2006, especially in rural areas. Hearing‐speech‐related co‐disabilities remained the dominant pattern, while mental‐related co‐disabilities increased most rapidly. The associations of different co‐occurring disability types with PD and moderate‐to‐severe PD varied. In the short term, priority should be given to identifying high‐risk co‐disability combinations and strengthening screening, referral, rehabilitation follow‐up, and continuity of care, particularly in rural areas. In the longer term, a more integrated co‐disability management framework is needed to better connect rehabilitation, mental health, primary healthcare, and long‐term care services. Greater use of the co‐disability rate indicator may also help guide priority setting and service planning.

## Author Contributions

**Yiran Wang:** conceptualization, methodology, formal analysis, writing – original draft, validation, funding acquisition. **Xiaodong Zhang:** conceptualization, methodology, formal analysis, writing – review and editing. **Lu Tan:** conceptualization, formal analysis, writing – original draft. **Chenyu Yan:** conceptualization, writing – review and editing. **Xiaoqian Yan:** writing – review and editing. **Wenwen Liu:** writing – review and editing. **Tianran Wang:** conceptualization, methodology, project administration, writing – review and editing, visualization, funding acquisition. **Sijiu Wang:** conceptualization, funding acquisition, supervision. **Wannian Liang:** conceptualization, funding acquisition, supervision.

## Ethics Statement

The present study used only de‐identified secondary data, involved no direct contact with human participants, and posed no more than minimal risk to participants. The original surveys were approved by the State Council of the People's Republic of China (Document No. 77 [1986] of the General Office of the State Council; Document No. 55 [2004] of the General Office of the State Council). The study protocols were conducted in accordance with the *Statistics Law of the People's Republic of China* and the *Law of the People's Republic of China on the Protection of Disabled Persons* and were compliant with the Helsinki Declaration and its subsequent revisions.

## Consent

Informed consent was obtained from all participants before data collection and clinical assessments in the original surveys. For the present secondary analysis, the authors used only fully de‐identified, pre‐existing survey data and had no access to personally identifiable information.

## Conflicts of Interest

The authors declare no conflicts of interest.

## Data Availability

The data that support the findings of this study are available from the Institute of Population Research of Peking University (IPR) upon reasonalbe request and with the permission of IPR. Restrictions apply to the availability of these data, which were used under license for this study.
